# Progress in the Development of SERS-Active Substrates Based on Metal-Coated Porous Silicon

**DOI:** 10.3390/ma11050852

**Published:** 2018-05-21

**Authors:** Hanna V. Bandarenka, Kseniya V. Girel, Sergey A. Zavatski, Andrei Panarin, Sergei N. Terekhov

**Affiliations:** 1 Applied Plasmonics Laboratory, Belarusian State University of Informatics and Radioelectronics, 220013 Minsk, Belarus; k.girel@bsuir.by (K.V.G.); sergeyzavatski13@gmail.com (S.A.Z.); 2 B.I. Stepanov Institute of Physics, National Academy of Sciences of Belarus, 220072 Minsk, Belarus; a.panarin@ifanbel.bas-net.by (A.P.); s.terekhov@ifanbel.bas-net.by (S.N.T.)

**Keywords:** porous silicon, metallic nanostructures, SERS, biosensing

## Abstract

The present work gives an overview of the developments in surface-enhanced Raman scattering (SERS) with metal-coated porous silicon used as an active substrate. We focused this review on the research referenced to SERS-active materials based on porous silicon, beginning from the patent application in 2002 and enclosing the studies of this year. Porous silicon and metal deposition technologies are discussed. Since the earliest studies, a number of fundamentally different plasmonic nanostructures including metallic dendrites, quasi-ordered arrays of metallic nanoparticles (NPs), and metallic nanovoids have been grown on porous silicon, defined by the morphology of this host material. SERS-active substrates based on porous silicon have been found to combine a high and well-reproducible signal level, storage stability, cost-effective technology and handy use. They make it possible to identify and study many compounds including biomolecules with a detection limit varying from milli- to femtomolar concentrations. The progress reviewed here demonstrates the great prospects for the extensive use of the metal-coated porous silicon for bioanalysis by SERS-spectroscopy.

## 1. Introduction

The surface-enhanced Raman scattering (SERS) effect is observed in noble metallic structures (SERS-active substrates) which have nanoscale roughnesses (10–150 nm) [1,2]. Since the discovery of this phenomenon in 1974 [3], approaches to the formation of the SERS-active substrates have undergone significant changes from the electrochemical roughening of metallic electrodes and the colloidal synthesis to various nanoengineering assemblies. An evolution of the fabrication techniques has been driven by the ever-increasing demand to improve the characteristics of the SERS-active substrates including the high enhancement factor, spot-to-spot and sample-to-sample reproducibility of the Raman signal, long shelf life, and the resistance to laser excitation and mechanical stresses [4,5]. In addition, it is very desirable to produce the SERS-active substrates, which are handy in practical use and have a reasonable price because they are often applied as expendable materials. To meet the requirement of the high enhancement factor, the SERS-active substrates have to be composed of arrays of nanoparticles (NPs) of noble metals with the position of the surface plasmon resonance (SPR) matching the excitation wavelength. The gaps between NPs must be about 2–10 nm to create a number of local sites with an extremely high electromagnetic field, so-called hot spots. The reproducibility of the SERS-signal intensity is provided by the fabrication of well-ordered arrays of metallic nanostructures of the same dimensions and shape. However, the production of the stable substrates is a rather complicated task because the nanoscaled objects are usually characterized by a very high surface energy and, in particular, silver and copper nanostructures have a tendency to reduce it via oxidation. To date, more than a hundred different types of the SERS-active substrates have been reported and they can be divided into two main groups: liquid colloidal substrates (metallic NPs in solution) and solid substrates (ordered arrays of metallic NPs on a planar substrate) [6].

Traditionally, the colloidal SERS-active substrates have been prepared by the chemical reduction of metal salts in a solution containing a special reducing agent [6]. Freshly synthesized NPs are stabilized due to the Coulomb repulsion of the similarly charged layer of ions (for example, citrate ions) on the particle surface. The alternative method of colloidal solutions fabrication is the laser ablation of metallic foil [7]. The resulting liquid substrates do not contain the waste products of the reactions. The properties of the NPs depend on the parameters of the laser and on the environmental conditions. The shortcomings of this method include the low productivity and a large size dispersion of the resulting particles (from 1–5 nm to 100–500 nm) [8]. There have also been other techniques of colloidal solution preparation developed. For example, plasma synthesis or thermolysis at which the metallic NPs are formed during the high-temperature decomposition of solids containing metallic ions, molecular anions, or organometallic complexes [9]. The undoubted advantage of the liquid substrates is the ability to objectively study the structure of long molecules such as DNA because the metallic NPs cover these molecules, preventing their deformation and allowing the registration of stable spectra [10], while the solid substrates cause changes in the conformation of molecules resulting in a lack or a shift of characteristic bands [11,12]. The liquid substrates demonstrate a high enhancement factor due to the many hot spots via NPs aggregation. Namely, the silver colloidal solution allowed the earliest single molecule detection by SERS-spectroscopy [13]. At the same time, aggregation leads to the drastic non-reproducibility and instability of the SERS-signal. From the point of view of the specific research in the conditions of scientific laboratories, these problems can be overcome since it is possible to renew the colloidal solution or to separate the particles, especially if steric stabilization is used [14]. However, if the SERS-study is performed by non-specialists who simply need to conduct routine measurements, the colloidal solutions cause difficulties. Thus, the solid substrates are more attractive for the wide practical applications.

The simplest way to obtain the solid SERS-active substrates is to dry the colloidal solution on the surface of glass, silicon plates, and so forth. This can be carried out in various ways, from drop-deposition to dipping the plate into a colloidal solution at a controlled rate. Unfortunately, such methods do not allow a good control over the resulting structure and the arrangement of the particles, including their plasmon properties. Other popular methods for the synthesis of the solid substrates are chemical and electrochemical deposition, thermolysis, ion bombardment, and the coarsening of silver foil with nitric acid [15]. Thanks to the development of nanotechnology, a large number of fundamentally new techniques for the fabrication of the solid SERS-active substrates have been proposed. Nanotechnology solves the above-mentioned problem of SERS-signal reproducibility because it is possible to create an ordered structure with control over the sizes, shapes, and spatial locations of the metallic nanostructures. Different nanolithography techniques are very popular for the fabrication of the solid SERS-active substrates. For instance, nanosphere lithography allows the synthesis of both ordered arrays of triangular NPs or metallized nanospheres [16,17], while ultraviolet or electron beam lithography can be used for the production of well reproducible SERS-active substrates based on nanoantennas, wires, rods, and nanostars [18,19,20]. Often these substrates are used for studying the SERS principles or application in different fields. In Reference [19], the thermal evaporation of gold following electron-beam lithography enabled the control of the metallic particle size, shape, and spacing. As a result, various elongated gold NPs were formed and used to locate the longitudinal SPR at the desired visible spectral range. This approach made it possible to optimize the Raman amplification up to 2 × 10^6^ per molecule. The other example of electron beam lithography for the growth of homogeneous arrays of cylindrical and ellipsoidal gold NPs is presented in Reference [20]. The obtained nanostructures were applied to understand the contribution of the localized surface plasmons to the enhancement of the Raman signal by so-called plasmon-sampled surface-enhanced Raman excitation spectroscopy. Later, in Reference [21], it was shown that linear gold nanoantennas fabricated by electron beam lithography on the CaF_2_ substrate are suitable for the combined SERS-SEIRS (surface-enhanced infrared scattering) study of molecules. The longitudinal dipolar resonance of 1–2 μm long antennas in the infra-red range was found to achieve SEIRS, while antennas with a 60 nm width and height showing the transverse SPR in the red region can be used for SERS-spectroscopy with a visible excitation wavelength.

By now a number of works on the alternative formation of the solid SERS-active substrates by templating them with porous materials have been published [22,23,24]. Under this approach, porous template plays the role of a host material, which takes the metal as a guest. In this way, the controllable definition of the dimensions and shapes of metal nanostructures is achieved. Materials such as porous alumina [22], porous silicon [23], and porous titanium oxide [24] can serve as a proper template. Among these materials, porous silicon takes a specific place due to several attractive features which include its well-controlled structure, rich morphological family, unique optical properties, the ability of ions of noble metals to be reduced on the surface of porous silicon via simple immersion deposition, and so forth. Using porous silicon as a template for the fabrication of the SERS-active substrates was first proposed in a patent application in 2002 [25]. The patent protected an idea to deposit metals into porous silicon layer instead of a standard flat substrate (glass) since the developed surface of the porous material is more favorable for the nucleation and growth of a greater number of metallic NPs. It should be noted that the authors of the patent proposed to cover the pore walls with a metallic film, that is, to realize metal deposition on the internal surface of the porous layer. An appropriate goal was to flow the analyte solution through the metal-coated porous silicon and register the SERS-spectra of the molecules adsorbed in the nanoscale pores. This patent application has initiated an intensive study and the development of the SERS-active substrates based on porous silicon template. Below we present a detailed review on the fabrication and the properties of porous silicon, its utilization as a template for the fabrication of the solid SERS-active substrates, and the problems and perspectives of porous silicon in the SERS-spectroscopy.

## 2. What is Porous Silicon?

The formation of porous silicon was first occasionally observed during the electrochemical polishing of silicon wafers in a hydrofluoric acid (HF) solution in 1956 by Arthur Uhlir and Ingeborg Uhlir who worked for Bell Lab [26]. This porous material is an artificial morphological form of silicon and possessed unique physical and chemical properties, which were determined by a network of nanosized pores in the crystalline matrix of silicon and the developed internal surface of these pores [27]. For a long time, porous silicon has been used only in its oxidized form as an insulating material in microelectronics [28]. However, in 1991, porous silicon experienced a new birth. It was discovered that the nanoscale silicon elements in porous silicon matrix possess quantum and surface effects [29]. Thanks to these features, porous silicon behaves as a direct-gap semiconductor demonstrating an intensive photoluminescence unlike monocrystalline silicon [29,30]. This phenomenon of porous silicon has been considered as an opportunity to create optoelectronic devices integrated with monocrystalline silicon [31]. A little bit later, porous silicon was found to act as a biocompatible and biodegradable material, provoking a new wave of its investigation for the medical purposes [32].

### 2.1. Fabrication of Porous Silicon

Porous silicon has been more often fabricated by anodic electrochemical etching of monocrystalline silicon in HF-based electrolytes [33]. The etching of silicon requires the presence of HF molecules (coming from the electrolyte) and holes (coming from the silicon wafer) at the reaction interface. To generate a sufficient number of electrons and holes in the silicon, its surface is often irradiated with light during the anodization process. This is especially necessary for the dissolution of *n*-type silicon and lightly doped *p*-silicon (*p*^−^-silicon).

The parameters of the porous layer, such as the porosity (the fraction of voids in the layer), thickness, size, and structure of the pores depend on the characteristics of the initial monocrystalline silicon and the anodizing regimes. The most important factors are the dopant type, the resistivity, and the crystallographic orientation of the silicon, as well as the concentration of HF, the pH level of the electrolyte, the presence of other compounds in it, the temperature, the current density, the illumination, the electrolyte mixing, and the duration of the anodic treatment. Optimal control of the fabrication of porous silicon and the reproducibility of the characteristics from process to process require the careful monitoring of these technological factors [33].

Some additional methods have also been utilized to form a template of a nanoscaled silicon skeleton for the SERS-active substrates. Reactive ion plasma etching [34,35,36,37,38,39,40,41,42,43,44,45], hydrothermal etching [46,47,48], and metal-assisted chemical etching (MACE) [49,50,51,52,53,54] are among these techniques. The common feature of the listed methods compared with electrochemical etching is in the formation of arrays of silicon nanowires. During the analysis of the papers devoted to SERS-spectroscopy with the metal-coated porous silicon, we have found that nearly 65% of SERS-active substrates based on porous silicon have been fabricated with the templates created by anodization (see Table S1). That is why, in the present review, we pay more attention to the substrates based on the anodically formed porous silicon.

### 2.2. Morphology of Porous Silicon

Porous silicon fabricated by anodic etching can be classified depending on the diameter and morphology of its pores [33].

*Microporous silicon* represents highly porous silicon sponges in which the pores are less than 2 nm in diameter. This morphology is typical for the porous layers formed in lightly doped silicon of both conductivity types (*n*^−^-Si, *p*^−^-Si) under external illumination. The pore diameters and sizes of the silicon nanocrystallites are self-regulating parameters during the fabrication of microporous silicon. Microporous silicon has been very rarely used for the fabrication of plasmonic structures due to its poor mechanical strength.

*Mesoporous silicon* is characterized by its 2–50 nm pore diameter while its porosity can reach 85–90%. It is mostly formed in highly-doped silicon (the specific resistance is less than 0.05 Ohm·cm) for both types of conductivity (*n*^+^-Si, *p*^+^-Si). In this case, the pores are ordered in slightly branched channels going perpendicularly from the silicon wafer surface to its volume. The porosity increase is achieved due to the etching of the small branches from the pore walls. A detailed description of the mesoporous silicon fabrication and structure can be found in Reference [55]. Mesoporous silicon has mostly been used as a host for the creation of ordered nanostructures of other materials [56,57]. This is caused by the highly ordered skeletons of the mesoporous silicon itself which defines the shape and sizes of the structures of the guest materials introduced into/onto the porous layer.

*Macroporous silicon* is usually formed on the lightly doped *p*- or *n*-Si. Silicon wafers of *n*^−^-type should be illuminated from the back side to generate the holes. The pore diameter varies in the range of several microns. The feature of this material is in the parallel cylindrical pores which are well-ordered. The porosity of macroporous silicon is rather low and typically does not exceed 20%.

All the types of porous silicon are characterized by highly developed surfaces. The pore walls have complicated morphology and are coated with a number of the broken bonds or surface states which anchor hydrogen ions and fluorine from the anodizing solution. Fresh porous silicon contains SiH_x_ and SiF_x_ (x = 1, 2, 3) [58]. The short air-storage leads to the oxidation of porous silicon and the formation of the O_x_–Si–H and O–i–O groups [59].

**Figure 1:**
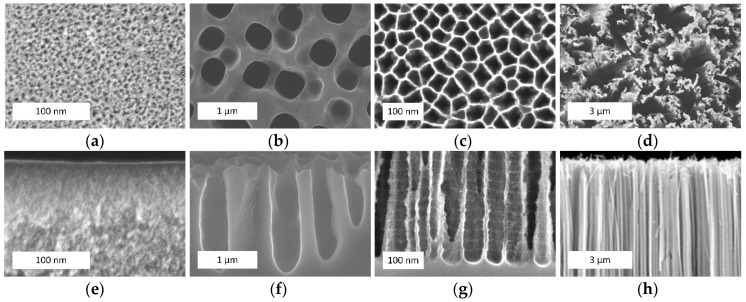
The scanning electronic microscopy (SEM) (**a**–**d**) top and (**e**–**h**) cross-sectional views of (**a**,**e**) microporous silicon; (**b**,**f**) mesoporous silicon; (**c**,**g**) macroporous silicon; and (**d**,**h**) silicon nanowires (provided by the authors).

Figure 1 shows examples of the different types of porous silicon formed by anodization (Figure 1a–c) and metal-assisted chemical etching (Figure 1d).

## 3. Why Porous Silicon is a Good Template for SERS-Active Substrates

Porous silicon has been considered a template for the formation of plasmonic materials, in particular, the SERS-active substrates due to the following reasons.

**Figure 2:**
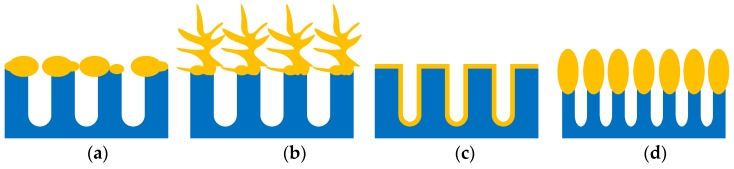
The schematic views of plasmonic structures formed on porous silicon: (**a**) metallic particles and (**b**) metallic dendrites on the outer surface of the porous layer; (**c**) conformal metallic film on the pore walls; (**d**) oval-shaped metallic NPs on the top of the silicon nanowires.

First of all its structure is easily managed by fabrication regimes and the type of the initial silicon substrates. This allows the definition of the morphology of the metallic nanostructures which are deposited on the porous layers. It is important that in order to have maximal SERS effect, a good matching between the excitation wavelength and the SPR band plays a crucial role. The latter strongly depends on the shape and size of the metallic nanostructures and the gaps between them, while porous silicon template helps to manage these properties. The family of the SERS-active nanostructures formed by metal deposition on porous silicon is very rich and includes metallic nanoparticles [60,61,62,63,64,65,66,67,68,69,70,71,72,73] or polydisperse particles [74,75,76,77,78,79,80,81,82], dendrites [49,54,83,84], nanovoids [85], and metallic oval-shaped NPs [37,38,39,40,41,42,43,44,45]. Figure 2 presents schematic views of plasmonic structures that can be formed with a dependence on porous silicon morphology, doping type, the level of silicon, and the method and conditions of metal deposition. Following Table S1, mesoporous silicon is the most popular template for the fabrication of the SERS-active substrates since it combines good mechanical strengths, nanoscale pores, and morphology reproducibility. The same benefits go from the silicon nanowires [50,51,53,73,86,87], nanotips [36], nanopillars [34,46,54,64], and so forth. Microporous silicon has not been used very often [88,89].

The SERS-active substrates formed on porous silicon have solid bases provided by the underlying silicon wafer. This is very important for the final user because this kind of substrates are favorable for practical applications as they can be diced into a few samples and their surface area can be easily defined during fabrication according to the customer’s request. Moreover, the silicon basis opens an opportunity to integrate the SERS-active substrates with other devices on the single silicon wafer.

It is well known that silver, gold, and copper (rarely) possess a high level of the Raman enhancement. At the same time, these metals are characterized by positive redox potential, which enables their deposition on the silicon templates from the water solutions of the corresponding metal salt by the immersion technique [90]. In this case, porous silicon acts as a source of a great number of electrons for the metallic atom reduction due to the developed surface area and dense surface states that appeared via breaking the monocrystalline structure [58,59]. Therefore, immersion deposition is a simple and inexpensive method resulting in the cost-effective fabrication of substrates that demonstrate outrageous SERS-activity.

**Figure 3:**
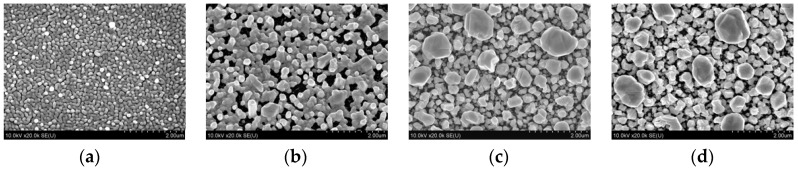
The SEM top views of (**a**,**b**) monocrystalline silicon and (**c**,**d**) mesoporous silicon coated with Ag NPs by immersion deposition. (**a**,**c**) as prepared samples, (**b**,**d**) samples kept in air at 21 °C for 3 months (provided by the authors).

**Figure 4:**
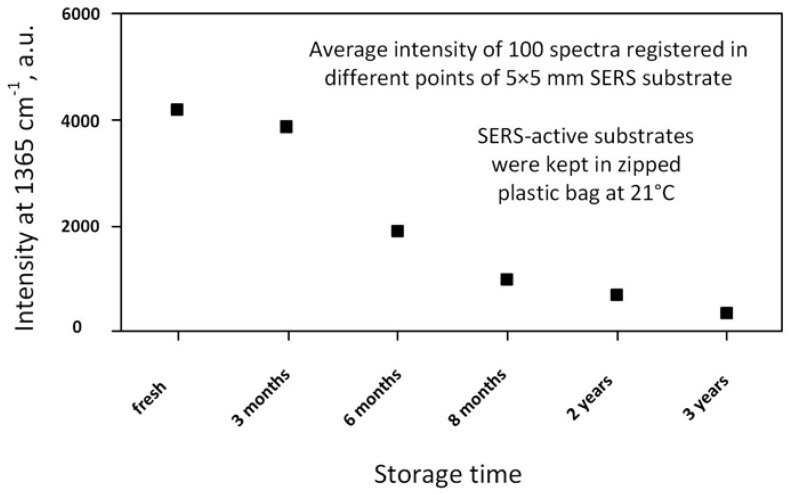
The SERS-intensity of 10^−6^ M R6G at the 1365 cm^−1^ band adsorbed on mesoporous silicon coated with Ag NPs by immersion deposition with a dependence on the time of the sample storage.

It has been found that porous silicon acts not only as a source of the electrons for the reduction of metal ions and as the template that defined the shape, sizes, and spatial arrangement of the metallic nanostructures, but also prevents their fast oxidation and coalescence, which usually leads to the inhibition of SERS-activity. For example, following Figure 3, one, can see that the morphology of the Ag coating on the silicon wafer is significantly changed after aging for 3 months resulting in the partial coalescence of the metallic NPs [91]. On the other hand, the as-prepared and aged samples of silver/porous silicon look the same. The energy dispersive spectroscopy of the presented samples revealed increases the oxygen atom content in the silicon-based samples up to 25.5% after aging while the silver/porous silicon samples were characterized by 5.5% oxygen content growth. To estimate the shelf life of the SERS-active substrates based on the silvered porous silicon, the SERS-spectra of 10^−6^ M rhodamine 6G (R6G) were registered. Figure 4 shows the dependence of the SERS-intensity of the 10^−6^ M R6G at the 1365 cm^−1^ band on the storage time of the silvered mesoporous silicon. The results of the measurements revealed the stability of the SERS-signal for 3 months. Moreover, the SERS-activity of the substrates was observed even for the samples aged for 2 years [91].

## 4. Approaches for Coating Porous Silicon with Metals for SERS

Plasmonic metallic nanostructures can be formed on porous silicon by different ways such as thermal decomposition [17,35,75], immersion deposition [74,77,83,89,92,93], deposition from colloids [64,68,94], evaporation [34,37], sputtering [36,37,53], physical vapor deposition (PVD) [95,96,97], pulsed laser deposition (PLD) [68,98], chemical deposition [99], and electrochemical plating [16,52,69]. However, here, we will only describe immersion deposition and thermal decomposition in detail. We have selected the immersion technique because it is the most popular for the metal coating of anodically grown porous silicon, which is of prevalent interest to this review. More precisely, about 60% of the SERS-active substrates based on porous silicon have been formed by immersion deposition (see Table S1).

Thermal decomposition deserves attention as it was one of the first reported methods to form plasmonic structures on porous silicon [17,25]. The deposition from colloids is not very common because the method usually leads to the non-uniform distribution of metallic NPs on the substrate and undesirable contaminations into the metal coating due to the by-products of colloids preparation. Electrochemical plating is limited by the fast rate displacement deposition on the silicon nanostructures typical for metals with a positive redox potential including silver and gold. This prevents the proper morphology control of metallic structures. In addition, the electrochemical deposition can lead to the formation of dense continuous metallic films on porous silicon without hot spots.

### 4.1. Thermal Decomposition of Metal Salt

In this method, silicon is soaked in a silver nitrate solution after exposure to a procedure of plasma oxidation to prevent the spontaneous immersion coating of the silicon surface [47]. The excess of the AgNO_3_ solution is removed from the pores using nitrogen gas and the sample is then dried to evaporate the solvent and adsorb the silver nitrate salt to the pore walls. Finally, the silver nitrate is thermally decomposed to silver metal and gases (NO_2_ and O_2_) by heating to 500 °C. It is applied to avoid complete filling porous silicon layer with metal but to form the continuous metallic coating of the pore walls. In order to take maximal advantage of the large surface area provided by porous silicon, the complete pore filling is undesirable for the SERS-active substrates because the useful surface area is reduced and the analytes are simply deposited on the top of porous silicon. It is known that just the silver coating has been formed by the thermal decomposition in porous silicon for the SERS-active substrates. In some works this method has been used as a final step after immersion deposition to provide the complete decomposition of the metal salt inside the pores [76,92].

### 4.2. Immersion Deposition of Metals

The immersion deposition of metals is the most popular technique to form SERS-active substrates based on porous silicon. The attractiveness of this method in comparison with the other wet techniques is in deposition of the metals on the substrate without applying an external potential or reducing agents to the solution. During immersion, the metallic atoms are deposited because of the oxidation–reduction reactions between the atoms of the substrate and the metallic ions from the solution. A remarkable coincidence takes place in the case of the silicon-based substrates. Namely, the copper and noble metals (silver, gold) that are the most favorable for the SERS-effect can be deposited on the silicon by the immersion method using the water solutions of the corresponding metal salt since these metals are characterized by their positive redox potential. The immersion of porous silicon into solutions containing the ions of these metals (Me^n+^) causes their reduction to the atomic form (Me_(solid)_) [83,90,93,100] to occur according to Equations (1)–(3):

2Si_(surface)_ + H_2_O → Si-O-Si_(surface)_ + 2H^+^_(aq)_ + 2e^−^ (1)

2Si-H_(surface)_ + H_2_O → Si-O-Si_(surface)_ + 4H^+^_(aq)_ + 4e^−^ (2)

Me^n+^_(aq)_ + ne^−^ → Me_(solid)_ (3)

By varying the dopant type and the doping level of silicon, the structure of porous silicon, and the regimes of the metal immersion deposition, one can reach partial or complete substitution of the silicon atoms in the porous layer with metallic atoms [93]. Following Table S1, we can see that different research groups have used various conditions of immersion deposition. Their results have shown that, in general, the morphology and, hence, the effectiveness of the plasmonic structures for SERS-spectroscopy mostly depend on two factors: the concentration of the metal salt in the immersion solution and the deposition time. The authors of Reference [101] found the parameter *effective time* critical for the management of the dimensions of the silver NPs and the gaps between them on mesoporous silicon. The *effective time* is calculated as a product of the AgNO_3_ concentration in the solution for immersion deposition by the silver deposition time. Choosing an optimal ratio between the concentration and time, that is, the proper value of *effective time*, provides the fabrication of the substrates resulting in the maximal SERS-activity.

## 5. Plasmonic Structures on Mesoporous Silicon

### 5.1. Substrates Based on p-Type Silicon

It should be noted that mesoporous silicon based on *p*-type silicon has been prevalently applied for the formation of the SERS-active substrates (Table S1).

In 2003, the authors of Patent [25] published an article on the investigation of the SERS-activity of silver-plated mesoporous silicon based on the *p*-Si, using R6G and adenine as analytes [17]. It was found that under excitation at 785 nm and with a laser power of 600 mW, the SERS-intensity rose with the increase of the porosity from 52 to 77%. The higher porosity led to the destruction of porous silicon. The detection limit of R6G reached during this work was 114 × 10^−9^ M. Later, the same group issued a number of patents covering methods of fabrication of the SERS-active substrates based on porous materials coated with the metallic NPs, including porous silicon [102], the detection of biological compounds using these substrates [103], and methods for the uniform coating of porous materials with noble metals [104].

In the work of Lin and coauthors [83], it was shown that the morphology of the nanostructured silver films strongly depends on the structure of porous silicon. The spontaneous growth of the silver dendritic structures made it possible to obtain a huge number of places with a strong local electromagnetic field (“hot spots”). It was revealed that during the preparation and further storage of the silvered porous silicon, various compounds were adsorbed on its surface. As a result, a strong background was observed in the SERS-spectrum, which overlaps the useful signal from the investigated substance. Therefore, the authors proposed a preliminary cleaning in diluted solutions of hydrochloric or sulfuric acids for several seconds. A study with an electron microscope showed that chemical cleaning led to the destruction of a certain amount of silver dendrites, which slightly inhibited the SERS-activity, but eliminated the undesirable background.

In Reference [74], a simpler method for the production of the SERS-active substrates based on porous silicon was proposed. Porous silicon was also formed by the electrochemical etching of *p*-type silicon. A nanostructured silver film was grown by immersion deposition from a water-alcoholic solution of silver nitrate. Silver particles of the nano- and sub-micron sizes located on the external porous silicon surface were observed. At the same time, a very small amount of the silver NPs was found inside the pores, in contrast to Reference [17] whose purpose was to cover the pore walls with a layer of silver.

A complex study of the properties of the SERS-active substrates based on the mesoporous silicon coated with silver NPs was carried out by Giorgis and coauthors in 2008 [92]. The SERS-active substrates were also formed by immersion deposition of silver on the electrochemically formed porous silicon, but with a subsequent annealing at 500 °C for the complete decomposition of silver nitrate, as well as for the removal of compounds adsorbed during the storage. The silver deposition was carried out for different periods, which allowed the growth of silver particles with sizes from 20 to 200 nm and chaotically located dendrites. In addition, the researchers moved away from the idea to cover the pore walls with a conformal silver film and focused on the deposition of silver nanostructures on the external surface of porous silicon. What is more, how the position of the SPR band depends on the silver deposition time by measuring the reflection spectra was shown. The reflection spectra were characterized by an absorption band at 315 nm associated with the bulk plasmons, as well as an additional band which is explained by the SPR arising from the individual particles and in places where the particles are closely located to each other. The resonance band can be expanded by forming a nanostructured film with a polydisperse particle size distribution. In a later paper [79] by Giorgis’s group, it was demonstrated that the use of an exciting laser with a wavelength close to the region of the SPR allowed the obtaining of maximum enhancement.

During the fabrication of the SERS-active substrates based on the metallized porous silicon grown on *p*-Si, not only the optimal conditions for the metal deposition were selected, but the porous silicon formation was also optimized, that is, the composition of the anodizing solution, the current density, and the anodizing time [105]. It was found that a change in the amount of HF in the etching solution leads to the formation of porous silicon with different structures, which greatly affects the morphology of the depositing silver. The maximum enhancement was achieved at the HF concentration of 25%, at which a close-packed silver film was formed further on the porous silicon surface. The authors explained this away by referring to a large number of S-H_x_ bonds in the pores that were involved in the reduction of silver.

In Reference [23], porous silicon was fabricated on *p*^+^-type silicon. The thickness of the porous layer was 6 μm while the pore diameter was about 15 nm. The silver was deposited from an aqueous solution of silver nitrate at different concentrations. The best result was achieved when a silver coating was formed in a 50 mM silver nitrate solution, in which silver aggregates up to 5 μm in size were formed. The authors suggested that the high SERS-activity observed with such samples can be associated with the adsorption of the analyte molecules in the gaps between the aggregates and the small NPs. The detection limits that were achieved on these substrates for R6G and crystal violet were 10^−12^ and 10^−8^ M, respectively.

**Figure 5:**
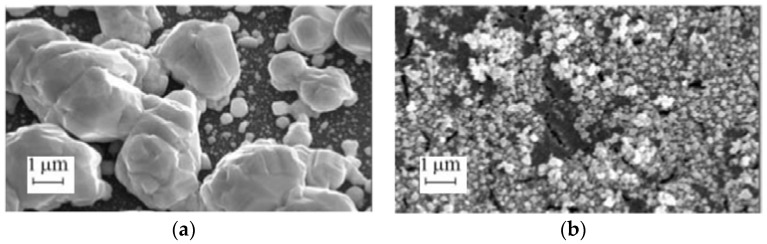
The SEM top views of porous silicon coated with silver for 10 min from the solutions with silver nitrate concentration of (**a**) 0.1 M and (**b**) 0.01 M.

In Reference [106], mesoporous silicon based on *p*^+^-type silicon (0.015 and 0.005 Ohm cm) was used to form the silvered SERS-active substrates. The silver was deposited on porous silicon from solutions with different concentrations of silver nitrate. It was found that the deposition of silver occurred predominantly at the mouths of the pores, whereas the pore walls were not covered with the silver NPs. The deposition from the most concentrated solution (10^−1^ M) led to the growth of large crystals of several to tens of microns in size (Figure 5). The use of the lower concentration of the silver nitrate (10^−2^ M) made it possible to obtain particles with a size of 100 ± 40 nm, which corresponds to the optimal particle size favorable for the SERS-effect. A maximum SERS-intensity was reached at a concentration of 10^−2^ M of silver nitrate and a deposition time of 10–15 min. The SERS-intensity was estimated at the 1650 cm^−1^ band in the copper porphyrin spectra. It was found that although the particle size arises with the increasing deposition time and the maximum of the plasmon resonance is expected to shift to the long-wavelength region, the position of the maximum in the SERS-intensity versus time graph did not change with the use of different lasers (457.9, 488, and 514.5 nm). The authors estimated the enhancement factor of the best SERS-active substrate. They used a method proposed in Reference [4] which exploited the already-known parameters for R6G, such as the fluorescence and the Raman cross sections. The calculated enhancement factor was 2 × 10^8^.

### 5.2. Substrates Based on n-Type Silicon

For the first time, porous silicon based on *n*^+^-type silicon was studied as a template for the SERS-active substrate in 2010 [107]. It was shown that the size and the number of silver particles on the surface of porous silicon strongly depended on the anodic current density. A low current density caused the formation of silver particles with sizes of 25–800 nm that were located at the great distance from each other. This suppressed the SERS-activity. The current density variation in the range 20–40 mA/cm^2^ led to the deposition of silver particles with an average size equal to 110 ± 4 nm. 

**Figure 6:**
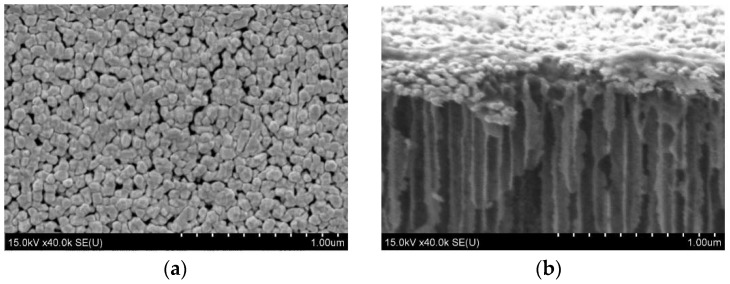
The SEM (**a**) top and (**b**) cross-sectional views of mesoporous *n*^+^-type silicon coated with gold NPs by immersion deposition (provided by the authors).

Many particles were densely packed, providing good conditions for “hot spots” and resulting in high SERS-activity. The samples obtained at a current density of 30 mA/cm^2^ demonstrated the highest sensitivity. Mesoporous silicon based on *n*^+^-Si is a very attractive material for the formation of the SERS-active substrates because it is characterized by highly ordered pore channels that are vertically grown in the silicon wafer. This feature results in a narrow range of size deviation for the further deposition of metallic particles and following a good reproducibility of the SERS-signal. The porosity of mesoporous *n*^+^-Si can reach 90%, that is, this material provides a great number of nucleation sites for the metallic particles. Additionally, the developed surface and electronic conductivity act as sources of electrons for the intensive reduction of the metallic ions during the immersion deposition. Figure 6 shows gold NPs on the surface of mesoporous silicon with a 70% porosity grown on *n*^+^-type silicon. It is well-seen that the particles have very similar sizes and are densely packed. Using R6G at a concentration of 10^−6^ M, we have found that the SERS-signal deviation across this gold-coated 5 × 5 mm substrate is about 5% at laser wavelengths of 633 and 785 nm. The diameter of the laser spot was about 860 nm for the red laser and 1064 nm for the infra-red one, while the mapping step of the substrate was 1 μm.

## 6. Plasmonic Structures on Macroporous Silicon

Macroporous silicon opens very wide opportunities for the SERS-active substrate fabrication. The pore depth of this porous material can be varied from less than one micron to several microns while the average pore diameter is more often applied as 1 µm.

The thick macroporous layer (more than several microns) is usually used to prevalently utilize its outer surface for the formation of plasmonic nanostructures. For instance, in Reference [108] macroporous silicon was used to grow gold thorns with a width of 50 nm and a length of 1 µm which allowed to reach the picomolar detection limit for the crystal violet. Paper [109] reported that the growth of silver dendrites on the top of macroporous silicon resulted in detection of CuTMpyP4 molecules at a concentration of 10^−10^ M.

Recently, macroporous silicon with a thickness of 38 µm, based on *n*^−^-Si was used as a template for the deposition of a layer of polydisperse gold particles [110]. These particles provided the detection of cyanine dye at a concentration of 10^−10^ M. The above-mentioned plasmonic structures were grown on macroporous silicon by immersion deposition.

Macroporous silicon has a pore diameter of more than 50 nm (up to several microns). Its pore depth also varied in the micrometer scale. Therefore, macroporous silicon can play a role of a template for the formation of so-called plasmonic nanovoids which are open cavities that are 1–2 µm in diameter in thin metallic films. They provide a significant Raman signal enhancement due to the localization of the surface plasmons of the strong ring component in the mouths of the voids and multiple light bounces inside the voids. Traditionally, the arrays of plasmonic nanovoids have been formed by nanosphere lithography [111]. The metal deposition on the macroporous silicon represents an alternative approach to create these peculiar nanostructures. The formation of the quasi-ordered array of bimetallic nanovoids onto macroporous silicon by successive nickel electroplating and silver immersion deposition was demonstrated in Reference [85]. The greatest increase of the SERS-signal was observed for the substrates containing 50 wt % silver and 5 wt % nickel, respectively.

The original structure based on macroporous SiO_2_ templates on a *p*-type silicon substrate with pore sizes of 500 nm was used to grow silver dendrites [112]. The silver deposition was carried out by immersion deposition from a solution of 0.02 M silver nitrate and 5 M at a temperature from 20 to 50 °C. Nile blue at a concentration of 10^−6^ M was used to study the Raman signal enhancement induced by Ag dendrites. Each macropore, in this case, played the role of the center for the silver dendrite nucleation.

An unusual approach that utilized macroporous silicon as a sacrificial template for the formation of a SERS-active copper porous film was reported in Reference [93]. The copper was deposited on a porous material from the HF-containing a solution of copper salt which resulted in the complete substitution of silicon atoms with those of the copper.

## 7. Application of PS-Based SERS-Active Substrates

The SERS-active substrates based on porous silicon make it possible to perform analyses of the wide range of analytes including the tetrapyrrolic molecules [74,84], proteins [94] and peptides [79,113], DNA [12,73], microRNA [62], gases [37,41,43,45], physiological fluids [97], thiols [95,114], and so forth. The femtomolar detection limit has been demonstrated for organic dye R6G [37,89,115], while the enhancement factor for some substrates can rise to an enormous value from 10^8^ [106,108,116,117] to 10^11^ [41].

### 7.1. Detection of Biomolecules

The paper by Lin and coauthors published in 2004 was one of the first works that reported on the detection of biomolecules with the SERS-active substrates based on porous silicon [38]. The silver dendritic structures were prepared on porous silicon by immersion plating and allowed for the adenine detection limit at 10^−9^ M.

It was shown in Reference [74] that the SERS-active substrates based on silver-coated porous silicon are efficient for the investigation of a number of water-soluble porphyrins H_2_TMpyP4, ZnTMpyP4, CuTMpyP4, and H_2_TPPS. Later, the same group was successful in the detection and study of the tetrapyrrole photosensitizer chlorin e6 [118].

Giorgis’s group has actively published papers on the fabrication of the SERS-active metallized porous silicon and its use for the detection of biomolecules at submolar concentrations, including the enzyme horseradish peroxidase [92] or oligopeptides [79,119]. They developed a detection approach from the immobilization of bioorganic molecules on the surface of the solid substrates made of silver NPs and porous silicon to the analyte flowing via the microfluidic system containing the silvered porous silicon membrane.

Recently, silvered mesoporous silicon based on *n*^+^-type silicon wafers were reported as effective SERS-active substrates which can be applied to the detection of DNA [12], phospholipids [115], and peptides, as well as for the reliable study of their secondary structure [113].

### 7.2. Antimony Detection

The detection and quantitative evaluation of antimony (III) using the SERS-active substrates made of silvered porous silicon were demonstrated in Reference [120]. The authors reported the method based on the analysis of the SERS spectra intensity of antimony bound to phenylfluorone (Sb-PhF), which is widely used as an organic reagent for the spectrophotometric determination of some heavy metals. The limit of Sb detection in the degassed samples reached 1 ng/mL. This concentration is one order of magnitude less than that attainable by the usually used photometric approach. One more advantage of the performed SERS-spectroscopy is in the very small sample volume (50 μL) required for the SERS analysis.

## 8. Limitations

Speaking about the limitations of porous silicon used as a template for the SERS-active substrates, we should take into account all the problems which appear for the porous media. In most cases, there are difficulties and doubts in the correct estimation of the enhancement factor and detection limit.

It is known that the enhancement factor is the ratio of the intensities of the scattered radiation for the SERS and Raman scattering per molecule. Most existing methods for the estimation of the enhancement factor are approximate and are not very suitable for porous SERS-active substrates. The main complication involves the determination of the number of molecules on the surface of the substrate within the area affected by the excitation radiation. The second problem is that for the correct determination of the enhancement factor, we need to use a monomolecular layer of the analyte, but it is complicated to detect the conventional Raman spectrum for such small amounts of the material. In addition, it is impossible to find the real number of molecules in the porous layer. To overcome this limitation, some modified approaches have been used to estimate the enhancement. For example, in Reference [79], the so-called external amplified Raman efficiency (EARE) was proposed. This parameter is defined as the ratio between the analyte concentration limit that is detectable on the metal-coated porous silicon with respect to the minimum concentration of the detected analyte adsorbed on the starting silicon substrate.

The same problem arises in relation to the detection limit. The analyte can be drop-deposited on the surface of the SERS-active substrates or adsorbed by immersion for a certain period. In both cases, we have no opportunity to objectively claim the detection limit. However, it is possible to say that we were able to detect the molecules deposited on the substrate from the solution at a given concentration.

The non-transparency in the visible range is one of the main problems typical for the solid SERS-active substrates based on the metal-coated porous silicon. It is impossible to correctly control the position of the SPR band because we cannot register the absorption spectra of the metallic nanostructures. The reflectance spectra give us doubtful information even in the study cases of full reflectance. 

The more specific limitation of porous silicon may be connected with its dissolution in the presence of alkaline liquids [121,122,123]. The solutions of some substances for SERS analysis can be prepared only at pH > 7. In Reference [120], it was found that silicon is significantly released from the porous silicon layer with a porosity ranging from 62 to 88% if impregnated with a solution at a pH that is higher than 6. The increase of the porosity, pH, and temperature from 25 to 37 °C resulted in better silicon dissolution. The later porous silicon layer thinning was observed during its storage in mineral water that had a pH = 7.2 and 8.1, at 18 °C [122]. Recently, the degradation of the SERS-active mesoporous silicon disks permeated with gold rods was observed at a pH of 7.4 [99]. The silicon skeleton was completely dissolved during the 8 h leading to the aggregation of the gold nanostructures and their loss of SERS-activity. However, the authors were able to slow down the porous silicon degradation to 24 h by modifying it with mPEG. At the same time, analyte solutions at a pH of 7.4 have been studied using the SERS-active substrates based on silvered mesoporous silicon [113] and gold-coated silicon nanopillars [38] but the silicon release was not observed. In this case, the metal coating was rather dense and prevented the contact of the solution and the silicon skeleton. Therefore, this negative effect can very likely take place if the analyte solution passes through the metallic nanostructures and directly into the porous layer.

## 9. Conclusions and Perspectives

The SERS-active substrates based on porous silicon have been used for the analysis of a wide range of analytes. The minimal concentration at which the analyte (R6G) has been detected with the use of substrates based on the anodically formed porous silicon reached 10^−15^ M [37,89,114]. Metal-coated silicon nanopillars showed minimal detection limit for the nerve gas VX equal to 13 × 10^−15^ M [45]. The enhancement factor for some substrates can reach 10^10^–10^11^ [17,41]. This value is well comparable to the value which is one of the best reported SERS-active substrates. Next, the important advantage of the substrates based on porous silicon is in their storage stability [91,108]. Some papers have also reported an extremely small spot-to-spot and sample-to-sample signal deviation: 14% [44] and 7% [124].

The prospects of porous silicon in the SERS-spectroscopy look very promising, especially for the microfluidic chips, which contain a SERS-active area of metallized porous silicon. This device can solve the problems of non-uniform distribution of analyte on the substrate and its impurities. In addition, the microfluidic chips based on porous silicon/PDMS membranes coated with metallic nanostructures were reported to successfully realize multianalyte detection [119].

Today, some SERS-active substrates based on mesoporous silicon and silicon nanopillars are commercially available [125]. Taking into account the very simple fabrication of porous silicon and its further covering with plasmonic metals, it can be suggested that the SERS-active substrates based on porous silicon can occupy a strong position among the signal improving materials for the SERS-spectroscopy in medicine, biology, forensics, pharmaceutics, analytical chemistry, and other areas which require the highly sensitive analysis of different substances.

## Supplementary Materials

The following are available online at http://www.mdpi.com/1996-1944/11/5/852/s1, Table S1: Analysis of works on the SERS-active substrates based on metal-coated porous silicon.
