# Supplementary material for: Progress in the Development of SERS-Active Substrates Based on Metal-Coated Porous Silicon
Source: Materials (Basel). 2018 May 21;11(5):852. doi: 10.3390/ma11050852 (PMC5978229; doi:10.3390/ma11050852)
Supplement: Supplementary file 1 [file materials-11-00852-s001.pdf]

Table S1. Analysis of works on the SERS-active substrates based on metal-coated porous silicon.

Number	Template type, parameters, fabrication method	Metal nanostructure type, parameters, method	Analyte, detection limit, excitation wavelength	Reference
		Microporous Si		
1	Microporous Si, *p*$^-$-Si, pore diameter < 2 nm, anodization	Ag NPs, diameter 30 nm, immersion, 10^{-2} M $AgNO_3$, 6 min	R6G, 10^{-9} M, 514.5 nm	[89]
		Mesoporous Si		
2	Mesoporous Si, *p*$^+$-Si, pore diameter 32 nm, porosity 52 – 77%, anodization	Ag NPs, oxidation, thermal decomposition, 10^{-3} M $AgNO_3$, 500°C	R6G, $1.14\cdot10^{-7}$ M, adenine, $9\cdot10^{-6}$ M, 785 nm	[17]
3	Mesoporous Si, *p*$^+$-Si, thickness 2 μm, anodization	Ag dendrites, immersion, 10^{-2} M $AgNO_3$, 5 min	R6G, 10^{-9} M Adenine, 10^{-9} M 488 nm	[83]
4	Mesoporous Si, *p*$^+$-Si, *p*-Si, anodization	Ag NPs, diameter 50 – 150 nm, immersion, 10^{-2} M $AgNO_3$, 10 min	tetrapyrrolic molecules H_2TMpyP4, ZnTMpyP4, CuTMpyP4, H_2TPPS, 10^{-6} M, 457.9, 532, 441.6 nm	[74]
5	Mesoporous Si, *p*$^+$-Si, thickness 1μm, anodization	Ag NPs, immersion, 10^{-3} M $AgNO_3$, 3 – 60 min, thermal decomposition, 500°C	Cyanine dye, 10^{-7} M, horseradish peroxidize, 10^{-8} M 514.5 nm	[92]

6	Mesoporous Si, *p*+-Si,<br>pore diameter 2-20 nm,<br>thickness 1 μm,<br>porosity 80%,<br>anodization	Ag particles,<br>1) immersion, 10^{-3} M – 10^{-2} M $AgNO_3$,<br>thermal decomposition, 500°C<br>2) oxidation, thermal decomposition<br>10^{-1} M $AgNO_3$	Cyanine dye, 10^{-7} M,<br>514.5 nm	[75]
7	Mesoporous Si, *p*+-Si,<br>pore diameter 20 – 40 nm,<br>anodization	Ag NPs + dendrites,<br>immersion, 10^{-2} – 10^{-3} M $AgNO_3$	CuTMpyP4,<br>10^{-6} M,<br>457.9 nm,	[84]
8	Mesoporous Si, *n*+-Si,<br>anodization	Au NPs,<br>diameters 4.5 nm, 14.8 nm<br>Colloidal on APTES	Benzenethiol, 10^{-3} M<br>785 nm	[114]
9	Mesoporous Si, *p*–-Si,<br>porosity 80 %,<br>anodization	Ag particles,<br>immersion,<br>10^{-3} M – 10^{-2} M, 50°C, 30 s – 10 min,<br>thermal decomposition, 500°C	Cyanine dye, 10^{-8} M,<br>514.5 nm	[76]
10	Mesoporous Si, *p*+-Si,<br>anodization	Au particles,<br>immersion, 10^{-3}, 10^{-2}, $5 \cdot 10^{-2}$ M $HAuCl_4$	R6G, $2 \cdot 10^{-5}$ M<br>633 nm	[77]
11	Mesoporous Si, *p*–-Si,<br>anodization	Ag particles,<br>ink jet printing, 10^{-2} M $AgNO_3$	Cyanine dye, 10^{-6} M,<br>514.5 nm	[78]
12	Mesoporous Si,<br>pore diameter 2 – 20 nm,<br>80% porosity,<br>anodization	Ag particles,<br>immersion 1 – 10 mM $AgNO_3$,<br>5 – 50°C, 30 s – 10 min	Cyanine dye, 10^{-8} – 10^{-9} M at 514.5 nm<br>CGIYRLRS peptide in ddH_2O, 10^{-3} M,<br>457.9, 514.5, 647 nm	[79]
13	Mesoporous Si,<br>pore diameter 15 nm,<br>thickness 6 μm,<br>anodization	Ag NPs,<br>immersion,<br>10^{-3}, 10^{-2}, $5 \cdot 10^{-2}$ M $AgNO_3$	R6G, 10^{-12} M at 514.5 nm,<br>crystal violet, 10^{-8} M at 633 nm	[23]

14	Mesoporous Si,<br>pore diameter 2 – 20 nm,<br>thickness 4 – 5 μm,<br>porosity 80 %,<br>anodization	Ag particles,<br>immersion,<br>best SERS for 10^{-2} M $AgNO_3$, 50°C, 60 s	Cyanine dye, 10^{-12} M, 514.5 nm<br>R6G, 10^{-7} M, 514.5 nm<br>R6G, 10^{-12} M, 647 nm	[80]
15	Mesoporous Si, p^+-Si,<br>pore diameter 15 nm,<br>thickness 3, 13 μm,<br>anodization	Ag particles,<br>diameter 10 nm – 5 μm,<br>immersion<br>10^{-2}, $5{\cdot}10^{-2}$ M $AgNO_3$, 120, 180, 210 s	R6G,<br>10^{-6} M,<br>532 nm	[81]
16	Mesoporous Si, p^-- Si,<br>thickness 1.7 μm,<br>porosity 64 %,<br>anodization	Ag NPs,<br>ink jet printing,<br>2.5, $5{\cdot}10^{-2}$ M $AgNO_3$	Cyanine dye, 10^{-6} M,<br>R6G 10^{-12} M,<br>514.5 nm	[117]
17	Mesoporous Si, p^+-Si,<br>pore diameter 30 nm,<br>thickness 4.8 μm, porosity 35 %,<br>anodization, oxidation	Au NPs/APTES,<br>diameter 5 nm,<br>colloidal deposition	Bovine serum albumine (BSA), 10^{-8} M,<br>488 nm<br>R6G, 10^{-8} M, 633 nm	[94]
18	Meso- on macroporous Si, p^--Si,<br>pore diameter of<br>meso- 10 – 30 nm,<br>pore diameter of<br>macro- 1 – 3 μm,<br>anodization	Ag NPs,<br>diameter 30 nm,<br>immersion,<br>10^{-2} M $AgNO_3$, 3 min	R6G,<br>10^{-15} M,<br>514.5 nm	[89]
19	Mesoporous Si, p^+-Si,<br>anodization	Ag NPs,<br>immersion, 10^{-2} M $AgNO_3$, 30 s	Crystal violet, 10^{-10} M,<br>785 nm	[60]
20	Mesoporous Si, *p*-Si,<br>pore diameter 10, 15, 30, 40 nm,<br>anodization	Ag NPs,<br>immersion,<br>10^{-1} M HF, 10^{-3}, $2{\cdot}10^{-3}$, $3{\cdot}10^{-3}$, $5{\cdot}10^{-3}$ M $AgNO_3$, 10 min	Malachite green, $2{\cdot}10^{-7}$ M,<br>532 nm	[61]

21	Mesoporous Si/PDMS membranes, *p*−Si, anodization	Ag NPs,<br>1) immersion, 10^{-2} M $AgNO_3$, 50 °C<br>2) ink jet printing, $2.5{\cdot}10^{-2}$ M AgNO3	1) ink jet, R6G 10^{-12} M, 514.5 nm<br>2) immersion, R6G, 10^{-12} M, 514.5 nm<br>3) immersion on PDMS supported membrane, R6G, 10^{-14} M, miRNA222, $485{\cdot}10^{-12}$ M	[16]
22	Mesoporous Si, *p*-Si, anodisation	Ag particles,<br>immersion, 10^{-2} – 10^{-3} M $AgNO_3$,<br>30, 60, 90, 120, 300 s.	R6G,<br>10^{-9} M,<br>514.5 nm	[82]
23	Mesoporous Si disks,<br>disk diameter 1 μm,<br>pore diameter 50 nm,<br>photolithography, anodization	Au nanorods,<br>diameter 6–10 nm,<br>length 30–50 nm,<br>chemical deposition	4-mercaptobenzoic acid,<br>10^{-8} M,<br>785 nm	[99]
24	Mesoporous Si disks, p^+-Si,<br>disk diameter 1 μm,<br>disk thickness 400 nm,<br>pore diameter 20 – 60 nm,<br>photolithography, anodization	Ag NPs/APTES,<br>immersion,<br>$4{\cdot}10^{-2}$ M $AgNO_3$	glutathione<br>(DTNB – Raman reporter),<br>$7.49{\cdot}10^{-8}$ M,<br>785 nm	[63]
25	Mesoporous Si grating, p^+-Si,<br>pore diameter 25 nm,<br>anodization, oxidation	Ag NPs/APTES,<br>diameter 30 nm,<br>colloidal deposition	R6G,<br>10^{-12} M,<br>785 nm	[64]

		Macroporous Si		
26	Macroporous Si, p^--Si,<br>pore diameter 0.5 – 1.5 μm,<br>anodization	Ag NPs,<br>immersion, 10^{-2} – 10^{-3} M $AgNO_3$	CuTMpyP4, 10^{-6} M,<br>457.9 nm	[84]
27	Macroporous Si, p-Si,<br>pore diameter 1.5 – 1.7 μm,<br>anodization	Au NPs (on Cr film, thickness 10 nm),<br>diameter 70 nm,<br>PVD	Thiol,<br>$2·10^{-3}$ M,<br>785 nm	[95]
28	Macroporous Si, p^--type,<br>pore diameter 0.5–1 μm,<br>anodization	Ag NPs/Ag colloidal NPs/APTES<br>immersion, Ag_2SO_4	R6G, $2·10^{-5}$ M, $2·10^{-8}$ M<br>633 nm	[65]
29	Macroporous Si, p^--Si,<br>anodization	Au NPs, diameter 50 nm,<br>PVD	11-Mercaptoundecanoic acid, $2·10^{-3}$ M,<br>785 nm	[66]
30	Macroporous Si, n^+-Si,<br>pore diameter 100 nm,<br>anodization	Au nanowires, length 300, 600, 900 nm<br>electrodeposition	10^{-4} M 4-4'bypiridine,<br>633 nm	[16]
31	Macroporous Si,<br>anodization	Au nanothorns on Au NPs,<br>nanothorns length 50 nm – 1 μm,<br>NPs diameter 5 – 20 nm,<br>immersion	Crystal violet, 10^{-15} M,<br>633 nm	[108]
32	Macroporous Si, n^--Si,<br>pore diameter 1.2 μm,<br>anodization	Ag NPs,<br>immersion	R6G, 10^{-9} M,<br>785 nm	[67]
33	Macroporous Si,<br>pore diameter 500 – 1500 nm,<br>anodization	Ag/Ni nanovoids,<br>Ni electrodeposition,<br>Ag immersion, 3 mM $AgNO_3$	CuTMpyP4, 10^{-6} M,<br>R6G, 10^{-11} M,<br>441.6 nm	[85]
34	Macroporous Si, p^--Si,<br>pore diameter 1.2 μm,<br>anodization	Ag dendrites on Ag NPs,<br>immersion,<br>$3·10^{-3}$ M $AgNO_3$, HF, C_2H_5OH, 80 min	CuTMpyP4,<br>10^{-10} M,<br>473 nm	[109]

35	Meso- on macroporous Si, *p*⁻-Si,<br>mesopore diameter 10 – 30 nm,<br>macropore diameter 1 – 3 μm,<br>anodization	Ag NPs,<br>diameter 30 nm,<br>immersion,<br>10^{-2} M $AgNO_3$, 3 min	R6G,<br>10^{-15} M,<br>514.5 nm	[89]
36	Macroporous Si, *p*⁻-Si,<br>anodization	Au, Ag NPs,<br>1) immersion, 10^{-2}, 10^{-3} M $AgNO_3$,<br>Ag NPs diameter 100 nm,<br>2) Ag colloidal deposition,<br>Ag NPs diameter 80 nm,<br>3) Au, Ag PLD,<br>Au, Ag NPs diameter 20 – 40 nm	1) Ag immersion,<br>R6G, 10^{-9} M, 514.5 nm<br>2) Ag colloid, R6G, 10^{-8} M, 514.5 nm<br>3) Ag, Au PLD, R6G, 10^{-8} M, 514.5 nm<br>Ag PLD, methylene blue, 10^{-10} M, 633 nm,<br>Au PLD, methylene blue, 10^{-8} M, 633 nm	[68]
37	Macroporous Si, *n*⁻-Si,<br>pore diameter 2 μm,<br>thickness 10 μm,<br>anodization	Au, Ag NPs,<br>electrodeposition,<br>$9 \cdot 10^{-3}$ M $HAuCl_4$, $9 \cdot 10^{-3}$ M $AgNO_3$	R6G,<br>10^{-3} M,<br>633 nm,	[69]
38	Macroporous Si, *n*⁻-Si,<br>anodization	AuNPs,<br>immersion,<br>10^{-2}, $5 \cdot 10^{-3}$ M $HAuCl_4$, 2.9 M HF, 3 min	Cyanine dye,<br>10^{-4}, 10^{-6}, 10^{-10} M,<br>514.5 nm	[110]
39	Macroporous Si, *p*⁻-Si,<br>SiO_2 thickness 250 nm,<br>pore diameter 500 nm,<br>anodization, oxidation	Ag dendrites,<br>immersion,<br>$2 \cdot 10^{-2}$ M $AgNO_3$, 5 M HF,<br>30 s, 20 – 50 °C	nile blue,<br>10^{-6} M,<br>473, 532, 633 nm	[112]

Porous Si type is not specified				
40	*p*-Si,<br>MACE,<br>2.5 M NH_4F, 10^{-2} M $AgNO_3$,<br>50°C	Ag dendrites,<br>grown during porous Si formation by MACE	Rhodamine B,<br>10^{-5} M,<br>633 nm	[49]
41	*p*-Si,<br>anodization	Ag NPs,<br>diameter 100 nm,<br>immersion, 10^{-3} – 10^{-1} M $AgNO_3$	R6G, 10^{-10} M, 514.5 nm<br>ZnTMpyP4, FeTSPP, 10^{-6} M, 457.9 nm	[106]
42	*p*-Si,<br>anodization	Ag NPs,<br>immersion, $AgNO_3$	chlorine e_6, 10^{-7} M,<br>457.9 nm, 514.5 nm,	[118]
43	*p*⁻-Si,<br>anodization	Ag NPs,<br>immersion	R6G, 10^{-5} M,<br>514.5 nm	[105]
44	*p*⁻-Si,<br>anodization	Ag NPs,<br>immersion, 10^{-2} M $AgNO_3$	ZnTMPyP4, 10^{-6} M,<br>441.6, 532 nm	[70]
45	*p*⁻-Si, anodization	Ag NPs, immersion	Sb-phenylfluorone complex, 532 nm	[71]
46	*p*⁻-Si,<br>anodization	Ag NPs,<br>diameter 50-100 nm,<br>immersion at ultrasonication,<br>best SERS at 10^{-2} M $AgNO_3$, 30 s	p-thiocresol, $5.2 \cdot 10^{-9}$ M,<br>2,4,6-trinitrotoluene, $1.1 \cdot 10^{-7}$ M,<br>633 nm	[116]
47	*p*⁺-Si,<br>anodization	Ag dendrites on NPs,<br>immersion,<br>10^{-2} M $AgNO_3$, 4 °C, 5 min. Sb	Sb-phenylfluorone complex,<br>limit of Sb detection 1 ng/mL,<br>514.5, 532 nm	[120]
48	*n*⁻-Si,<br>1) light-induced etching,<br>2) anodization	Ag NPs,<br>immersion, 10^{-2} M $AgNO_3$, 15 min	R6G, 10^{-15} M,<br>514.5 nm	[72]
49	*p*⁺-Si[1],<br>anodization	Au film,<br>thickness 10, 30, 50, 100, 200, 300 nm,<br>PVD	p-mercptobenzoic acid, 10^{-2} M,<br>human blood, cerebrospinal fluid, urine,<br>785 nm	[97]

		Si nanotips, nanowires, nanopillars		
50	Poly-Si nanopillars, height 0.1 – 0.3 μm, diameter 20 – 100 nm, reactive ion etching	Au layer, thickness 10 – 20 nm, evaporation	4',6-diamidino-2-phenylindole (DAPI), 10^{-3} M, 785 nm	[34]
51	Poly-Si nanopillars, height 250 nm, diameter 40 nm, reactive ion etching	Ag film, thickness 50 nm, oxidation, thermal decomposition	R6G, 10^{-9} M, 785 nm	[35]
52	Si nanotips, height 1 μm, diameter 2 (top) – 100 (bottom) nm, reactive ion etching	Ag NPs, diameter 4 – 10 nm, ion beam sputtering	R6G, trans-1,2-bis(4-pyridyl)ethylene (BPE) 10^{-6} – 10^{-10} M, 532 nm	[36]
53	Si nanowires, MACE, $AgNO_3$/HF, HF/$Fe(NO_3)_3$	Ag film/colloidal Au NPs/ 3-aminopropyltrimethoxysilane (APTMS), immersion	R6G, $5{\cdot}10^{-7}$ M, amoxicillin , 10^{-6} – 10^{-9} M, calcium dipicolinate, $4{\cdot}10^{-6}$ M, 514.5 nm	[50]
54	Si nanowires, p^{-}-Si, MACE, vapor liquid (VLS)	Ag NPs, diameter 4 – 40 nm, immersion, 10^{-3} M $AgNO_3$, 0.26 M HF, 60s	R6G, For VLS substrates 10^{-14} M For MACE substrates 10^{-9} M 633 nm	[51]
55	Nanoporous Si pillars, hydrothermal etching	Ag NPs, diameters 30 – 120 nm, 400 – 1000 nm, immersion, 10^{-2} M $AgNO_3$, 5 min	Adenine, 10^{-4} – 10^{-6} M, 532 nm	[46]
56	Nanoporous Si pillars, p^{+}-Si, hydrothermal etching	Cu NPs, immersion, 10^{-3} M $CuCl_2$	R6G, 10^{-5} M, 633 nm	[47]

57	Si nanowires,<br>oxide-assisted growth via thermal evaporation of Si monoxide	Ag NPs,<br>immersion, drop of 1 M $AgNO_3$ after 10^{-1} M NaOH, 15 min	DNA 10^{-15} M,<br>633nm	[73]
58	Si nanopillars,<br>length 600 – 1600 nm,<br>gap between pillars 50 – 80 nm,<br>reactive ion etching	1) Oval-shaped Ag NPs,<br>electron-beam evaporation<br>2) Ag coating,<br>magnetron sputtering	Thiophenol gas,<br>BPE, 10^{-3} M<br>785 nm	[37]
59	Porous Si pillars, p^+-Si,<br>hydrothermal etching	Ag particles,<br>diameters 70 nm, 100 – 1000 nm,<br>immersion,10^{-2} M $AgNO_3$, 1 – 10 min	532 nm<br>R6G, 10^{-15} M	[48]
60	Si nanopillars,<br>nanosphere lithography,<br>wet chemical etching, MACE	Ag coating,<br>electrodeposition,<br>$AgNO_3$, H_2BO_3	R6G, 10^{-6} M,<br>488 nm	[52]
61	Si nanowires, *p*-Si,<br>MACE	Au film,<br>thickness 30 – 300 nm,<br>sputtering	1,2-benzenedithiol (BDT), 10^{-5} M,<br>785 nm	[53]
62	Si nanopillars, undoped Si,<br>height 750 nm,<br>gap between pillars 50 – 80 nm,<br>reactive ion etching	Oval-shaped Au NPs,<br>length 250 nm,<br>aptamers for detection of analyte,<br>electron-beam evaporation	TAMPA-labeled vasopressin (TVP),<br>10^{-12} M,<br>633 nm	[38]
63	Si nanopillars, undoped Si,<br>height 750 nm,<br>gap between pillars 50 – 80 nm,<br>reactive ion etching	Microfluidic device,<br>oval-shaped Au NPs,<br>length 250 nm,<br>aptamers for detection of analyte,<br>electron-beam evaporation	TVP, $2{\cdot}10^{-10}$ M,<br>633 nm	[39]
64	Si nanopillars,<br>height 400 nm,<br>gap between pillars 200 nm,<br>reactive ion etching	Ag oval-shaped NPs,<br>diameter 62 nm,<br>evaporation	covalent diphenylalanine nanotube-folic acid conjugates,<br>780 nm	[40]

65	Si nanopillars, undoped Si, reactive ion etching	Oval-shaped Ag NPs, electron-beam evaporation	ethanol vapor, 0.0017 ng, acetone vapor 0.0037 ng, 633 nm	[41]

66	Si nanopillars, height 600 nm, width 50 nm, reactive ion etching	Oval-shaped Ag NPs, height 300 nm, width 120 nm, electron-beam evaporation	Folic acid, 10^{-9} M, 532nm	[42]
67	Si nanopillars, height 400 nm, width 50 nm, reactive ion etching	Oval-shaped Au NPs, evaporation	HCN gas, 5 ppm, KCN liquid, 18 ppb (10^{-5} M), 780 nm	[43]
68	Si nanopillars, p^{-}-Si, reactive ion etching	Ag NPs (on Cr film, thickness 3 nm), electron beam evaporation	BPE, 10^{-11} M, 780 nm	[44]
69	Si nanopillars, reactive ion etching	Oval-shaped Au NPs, evaporation	nerve gases VX, $1.3\cdot10^{-14}$ M, Tabun, $6.7\cdot10^{-13}$ M, 785 nm	[45]
70	Oxidized porous Si, p^{+}-Si, anodization	Au NPs, diameter 30 nm, PVD	R6G, 10^{-6} M, 633 nm	[96]
71	Si nanowires, CVD	Au NPs, immersion, CVD of graphene	R6G, 10^{-6} M, 532, 785 nm	[87]
72	Si nanopillars, p-Si, height 10 μm, MACE	Ag dendrites, immersion, 10^{-2} M $AgNO_3$, 4.6 M HF, 120 s	thiophenol, 10^{-2} M, mixture of R6G, methylene blue, 10^{-4} M, 785 nm	[54]
